# Lessons from two prevalent amyloidoses—what amylin and Aβ have in common

**DOI:** 10.3389/fnagi.2013.00038

**Published:** 2013-08-08

**Authors:** Jürgen Götz, Yun-An Lim, Anne Eckert

**Affiliations:** ^1^Centre for Ageing Dementia Research, Queensland Brain Institute, The University of QueenslandBrisbane, QLD, Australia; ^2^Sydney Medical School, Brain and Mind Research Institute, University of SydneySydney, Australia; ^3^Memory, Aging and Cognition Centre, National University Health SystemNational University of Singapore, Singapore; ^4^Neurobiology Laboratory, Psychiatric University Clinics Basel, University of BaselBasel, Switzerland

**Keywords:** ABAD, Alzheimer's disease, amylin, amyloid, diabetes, mitochondria, proteomics, vaccination

## Abstract

The amyloidogenic peptide Aβ plays a key role in Alzheimer's disease (AD) forming insoluble aggregates in the brain. The peptide shares its amyloidogenic properties with amylin that forms aggregates in the pancreas of patients with Type 2 Diabetes mellitus (T2DM). While epidemiological studies establish a link between these two diseases, it is becoming increasingly clear that they also share biochemical features suggesting common pathogenic mechanisms. We discuss commonalities as to how Aβ and amylin deregulate the cellular proteome, how they impair mitochondrial functions, to which receptors they bind, aspects of their clearance and how therapeutic strategies exploit the commonalities between Aβ and amylin. We conclude that research into these two molecules is mutually beneficial for the treatment of AD and T2DM.

## Introduction

A rapidly ageing population and the modern sedentary lifestyle, combined with changes in diet, have to be blamed for the fact that both Type 2 Diabetes mellitus (T2DM) and Alzheimer's disease (AD) are reaching epidemic proportions. World-wide numbers of patients with diabetes are projected to rise from ~171 million at the turn of this century to 366 million by 2030 (Wild et al., 2004). For comparison, more than 26 million people are currently living with AD, a number that will quadruple to more than 106 million by 2050 unless strategies are put into place that slow down, cure or even prevent dementia altogether. Of all dementing disorders, AD is the most common form, comprising 50–70% of all cases (Ferri et al., 2005). AD is characterized by impaired memory, visuospatial, language, and executive functions. While cognitive deficits have traditionally been emphasized in defining AD, there are a variety of neurobehavioral symptoms also commonly associated with the disease, including increased apathy, agitation, anxiety, and psychiatric symptoms such as delusions or hallucination (Assal and Cummings, 2002).

In the AD brain, there are two key molecules that undergo a change in tertiary structure followed by self-association and deposition, the amyloid-β (Aβ) peptide and tau, a microtubule-associated protein (Götz and Ittner, 2008). Aβ is proteolytically derived from the amyloid precursor protein, (APP), and is the major constituent of Aβ plaques; its size (ranging from 37 to 43 amino acids) is modulated by the thickness of the plasma membrane at the site where the peptide is generated (Area-Gomez et al., 2012; Winkler et al., 2012). While increased levels are detrimental, in a physiological context, Aβ has been postulated to have a role in learning and memory (Morley et al., 2010). Hyperphosphorylated tau is the major constituent of neurofibrillary tangles (Iqbal et al., 2010). Physiological functions of tau include the binding and stabilization of microtubules in the axon, and more recently, targeting of the kinase Fyn to the dendrite or protecting the DNA in the nucleus from various forms of insult (Ittner et al., 2010; Sultan et al., 2011). What is causing the majority of cases of AD is not known. In the elucidation of disease mechanisms, rare familial cases were instrumental. In AD, mutations have been identified in the (*APP*) gene itself, as well as in the genes that encode presenilin 1 and 2 (*PSEN1* and *PSEN2*), a protein forming part of the Aβ-generating protease complex. Frontotemporal lobar degeneration (FTLD) is the second-most prevalent form of dementia. This complex is classified based on the proteins that constitute the major brain aggregates. FTLD-Tau, for example, is characterized by tau aggregates. These are found in the absence of overt Aβ plaque formation. Again, there are rare familial forms, for which disease-causing mutations have been identified in the *MAPT* gene that encodes tau (Lee et al., 2001). AD is predominantly characterized by memory loss, whereas FTLD is mainly initiated by behavioral impairment, with cognitive functions relatively preserved until the disease becomes more advanced. The neurobehavioral symptoms include overeating, apathy or euphoria, disinhibition, depression, stereotyped behaviors, reduced empathy, and antisocial and aggressive behaviors. Patients with FTLD also display a variety of cognitive problems, such as language and memory impairments, and these are often coupled with a lack of insight into these changes (Neary et al., 1998). In a significant subset of FTLD, late Parkinsonism is found (Lee et al., 2001). To better understand the role of Aβ and tau in AD and FTLD, experimental mouse models have been developed, in particular in mice, that reproduce the major aspects of the neuropathological characteristics of these diseases, along with memory and motor impairment (Götz and Götz, 2009).

Diabetes mellitus (DM) is subdivided into Type 1 (T1DM) and Type 2 (T2DM), with the latter accounting for 90% of all cases. T1DM is characterized by reduced insulin production due to the destruction of pancreatic islet β-cells, whereas T2DM is characterized by insulin resistance of the target tissue, both of which results in elevated blood glucose levels. Disease progression correlates well with amylin deposition, a peptide with a central role in the control of energy homeostasis and satiety that under normal conditions functions as a synergistic partner of insulin (Lutz, 2010). Interestingly, amylin, that is also known as Islet Amyloid PolyPeptide (IAPP), forms aggregates already in the pre-diabetic stage. In doing so it undergoes a change in tertiary structure, similar to what is known for Aβ and tau in AD, and the peptide is finally deposited in β-cells (Hoppener et al., 2000), becoming a characteristic histopathological hallmark lesion of T2DM (Marzban et al., 2003; Hoppener and Lips, 2006). In humans, amylin is synthesized with a 22 amino acid signal peptide that is cleaved off resulting in an inactive 67 amino acid-long propeptide that is colocalized with insulin in β-cell granules. The mature 37-amino acid peptide is then generated by proteolysis that employs a set of proteases. When there is too much amylin this causes an impairment of glucose-mediated insulin secretion and ultimately β-cell death, as shown both *in vitro* and *in vivo* (Tokuyama et al., 1997; Hoppener et al., 2000; Hoppener and Lips, 2006).

For T2DM, the discovery of causal genes has followed three waves as discussed recently (McCarthy, 2010): The first wave consisted of family-based linkage analysis and focused candidate-gene studies. This proved effective in identifying the genes encoding leptin, the leptin receptor and proopiomelanocortin in extreme forms of early-onset disease that segregate in a Mendelian manner as single-gene inherited disorders. The second wave of discovery involved a switch to association studies. Common risk-conferring coding variants were identified in *PPARG* (encoding peroxisome proliferator-activated receptor gamma), *KCNJ11* (potassium inwardly-rectifying channel, subfamily J) and *MC4R* (melanocortin-4 receptor). The third wave of discovery has been driven by systematic, large-scale surveys of the association between common DNA sequence variants and disease. This identified an association between T2DM and variants within *TCF7L2* (encoding transcription factor 7–like 2, a protein not previously identified as a candidate) (Grant et al., 2006). TCF7L2 has subsequently been shown to modulate pancreatic islet function (Lyssenko et al., 2007). For amylin itself, promoter variants and specific mutations such as Ser20Gly have been shown to be associated with T2DM in some studies, whereas others showed no association, including one study with over 22,000 participants (Zee et al., 2011).

Similar to AD, T2DM has been modeled in mice: for example, in the ob/ob (leptin knockout) and db/db (leptin receptor knockout) strains, both of which are insulin resistant (Zhang et al., 1994; Chen et al., 1996), and in mice that over-express human amylin in pancreatic islet cells (de Koning et al., 1994; Janson et al., 1996; Verchere et al., 1996; Westermark et al., 2000). Breeding of amylin transgenic mice to homozygosity caused amylin aggregation, β-cell death and diabetes (Janson et al., 1996). One study showed that transgenic overexpression of fibrillogenic human amylin in mice caused β-cell degeneration and diabetes by mechanisms independent of both peripheral insulin resistance and islet amyloid. These findings are consistent with β-cell death evoked by misfolded but soluble cytotoxic species, such as those formed by human amylin *in vitro* (Wong et al., 2008). Similarly, transgenic rats with β-cell overexpression of human amylin developed islet amyloid deposits; these were associated with β-cell death and hyperglycemia (Matveyenko and Butler, 2006a). Diabetes can also be induced *in vivo* by injecting streptozotocin or by allogeneic expression of major histocompatibility antigens, and both causes the destruction of β-cells (Götz et al., 1990; Rees and Alcolado, 2005).

The basis of non-pharmacological DM treatment is diet and physical activity. About one third of T2DM patients need insulin to reduce their high blood glucose levels, but they do not critically depend on the hormone (Harrison et al., 2004; Hussein et al., 2004). In comparison, the current treatment of AD is symptomatic, slowing the cognitive decline only moderately; FDA-approved treatments include acetylcholine esterase inhibitors, and the NMDA glutamate receptor antagonist Memantine that counteracts excitotoxicity (Ballard et al., 2011).

In conclusion, both T2DM and AD are characterized by insoluble protein aggregates with a fibrillar conformation—amylin in T2DM pancreatic islets, and Aβ and tau in AD brain (Götz et al., 2009). Amylin aggregation is associated with pancreatic β-cell loss (although T2DM is believed to arise from insulin resistance in target organs), whereas Aβ and tangle formation is associated with neuronal cell loss. β-Cell loss leads to diabetes, nerve cell loss to dementia. Therefore, T2DM and AD are both conformational diseases.

### Comparison of human amylin, rat amylin and Aβ

**Figure 1:**
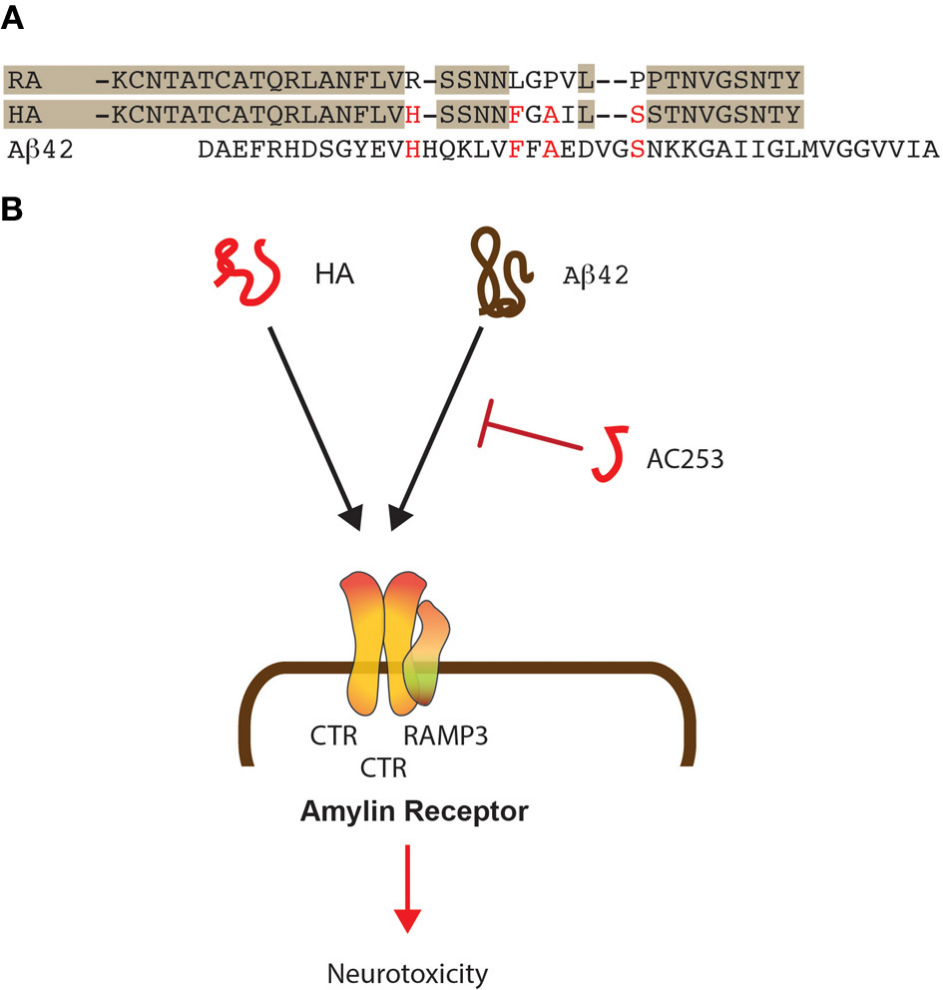
**Shared mode of toxicity of human amylin and Aβ42. (A)** Alignment of the sequences of human (HA) and rat amylin (RA) and comparison to Aβ 42. Shading shows similarities in the rat and human amylin sequences while important similarities between HA and Aβ 42 are shown in red. **(B)** HA and Aβ 42 both bind to the amylin receptor that is composed of two molecules of CTR and one molecule of RAMP3. The small HA-derived peptide AC253 abrogates toxicity of both HA and Aβ 42 mediated via the amylin receptor.

Human amylin shares many biophysical and physiological properties with Aβ (Figure 1A). The two molecules are similar in size but have little similarities in their primary sequence. However, different from rat amylin, they fold into similar secondary structures. In an attempt to convert the 37 amino-acid human amylin into a less amyloidogenic and hence, less toxic molecule, site-directed mutagenesis has been performed; this identified residues 13 and 15–17 as an additional aggregation-prone region outside of the established amyloidogenic region that comprizes residues 20–29 (Fox et al., 2010). Molecular dynamics (MD) simulations showed that within this region, Pro28 in rat amylin influences the secondary structure by stabilizing the peptide in a helical conformation, whereas human amylin (that has a serine at this position) is highly disordered in the carboxy-terminal region, presenting transient isolated β-strand conformations. These conformational differences are likely responsible for the different aggregation propensities of the two homologues. As fragment 30–37 known to aggregate *in vitro* is identical in both homologues, it has been argued that the overall sequence must be responsible for the amyloidogenic properties of human amylin, whereas the increased helicity in rat amylin due to a proline rather than a serine at position 28 may explain why it is not forming aggregates (Andrews and Winter, 2011). Interestingly, by dissolving lyophilized rat amylin directly in 20 mM Tris-HCl (and not first in water), rat amylin is in fact capable of forming fibrils. These structures bind the amyloidogenic dye Congo red indicating that they are real fibrils. Rat amylin fibrils generated by this method are toxic to both pancreatic islet and neuronal cell culture systems, presenting them as a novel anti-amyloid drug discovery tool (Milton and Harris, 2013).

In amyloidoses, the proteins in question are subjected to posttranslational modifications. Of the non-enzymatic posttranslational modifications, deamidation of asparagine and glutamine is the most common. Deamidation can influence the structure, stability, folding, and aggregation of proteins and has been proposed to play a role in amyloid formation in AD (Shimizu et al., 2005). While the role deamidation has in AD in forming amyloid plaques is not fully understood, one hypothesis is that in the turn region of Aβ, the Asp23Asn mutation and subsequent deamidation to isoAsp23 might cause a structural change that subsequently initiates folding of Aβ into β-sheets (Sargaeva et al., 2009). By examining the effects of deamidation on the kinetics of amyloid formation by amylin it was found that deamidation accelerates amyloid formation and that the deamidated material can seed amyloid formation of unmodified amylin (Dunkelberger et al., 2012). This is highly reminiscent of what has been reported for fibrillar tau that was found to recruit “normal” tau into aggregates (Guo and Lee, 2011), and interestingly, tau is also a deamidated protein (Watanabe et al., 1999).

### The amylin receptor as a mediator of Aβ toxicity

For the two amyloidogenic molecules amylin and Aβ, shared and separate modes of toxicity have been revealed, that are in part receptor-mediated, with the receptors, at least to some degree, being shared between the two molecules (Götz et al., 2008). One of these receptors is the amylin receptor that exists as a dimerized form of the calcitonin receptor (CTR) complexed with the Receptor activity modifying protein 3 (RAMP3), all of which are highly expressed in brain (Yamin et al., 1994; Ueda et al., 2001). This trimeric constellation generates a receptor that binds amylin with a significantly higher affinity than, for example, Calcitonin gene related peptide (CGRP) or adrenomedullin (Young, 2005) (Figure 1B). As discussed below, Aβ toxicity in rat cholinergic basal forebrain neurons can be blocked by the amylin receptor antagonist AC187 (Jhamandas and MacTavish, 2004). Another receptor that is shared between Aβ and amylin is APP, and both molecules induce APP expression in neuronal and astrocyte cultures (White et al., 2003). The neurotoxicity of the two molecules is furthermore mediated by specific integrin signaling pathways, and both can be inhibited with integrin-specific antibodies and cytochalasin D (Wright et al., 2007).

We found by quantitative proteomics that human amylin and Aβ deregulate identical mitochondrial proteins supporting the notion that both amyloidoses have common targets (Lim et al., 2010). The human amylin receptor mediates the biological effects of Aβ as shown by the team of Jhamandas et al. (2011), and in Aβ-forming transgenic mice, the receptor is up-regulated in brain regions with an elevated amyloid load (Jhamandas et al., 2011). Patch clamping of human foetal neurons showed that the electrophysiological effects of Aβ could be blocked with yet another amylin receptor antagonist, the highly selective 24 amino acid-long AC253 (Jhamandas et al., 2011). AC253 attenuated Aβ-mediated caspase-dependent and -independent apoptotic cell death in human foetal neurons (Figure 1B). In Aβ-forming mice, both amylin receptor subunits were found up-regulated in brain areas with a high amyloid burden, suggesting that the latter may be a signal for up-regulation. In a follow-up study, depression of hippocampal LTP in Aβ-forming transgenic mice was found to be mediated through the amylin receptor (Kimura et al., 2012). Pre-application of AC253 blocked both Aβ- and human amylin-induced reductions of LTP without affecting baseline transmission or LTP on its own. The data were complemented by field EPSP recordings in hippocampal slice cultures of wild-type mice; in these, Aβ and human amylin depressed the induced LTP, whereas AC253 enhanced the blunted LTP. Using rat und human neuronal cultures, application of either Aβ or human amylin, but not rat amylin, caused a time-dependent increase of apoptosis markers at the transcriptional level (Jhamandas and Mactavish, 2012). When AC253 was added following exposure to Aβ 42 or human amylin, this attenuated the induction of several pro-apoptotic mediators and increased the expression of anti-apoptotic markers. Together, this suggests that Aβ toxicity is, at least in parts, mediated by the amylin receptor.

### Effects of elevated glucose levels in alzheimer models

Compelling evidence indicates that excess consumption of sugar-sweetened beverages and high fat diets play an important role in the epidemic of obesity, a major risk factor for T2DM, a disease that has been associated with a higher incidence of AD. When Aβ plaque-forming transgenic APP/PS1 mice were fed with 10% sucrose-sweetened water, compared with control mice fed with no sucrose added to the water, the sucrose group gained more body weight and developed glucose intolerance, hyperinsulinemia, and hypercholesterolemia. These metabolic changes were associated with an exacerbation of the memory impairment that characterizes the Aβ-plaque-forming model, and an up to 3-fold increase in insoluble Aβ levels and its deposition in the brain. Interestingly, steady-state levels of IDE did not change, but there was a 2.5-fold increase in brain apolipoprotein E levels, a molecule with a central role in sporadic AD (Cao et al., 2007). In a complementary study, diabetic BBZDR/Wor rats (T2DM model) were assessed for characteristic AD changes in their frontal cortices. While neuronal loss was also found in a model of T1DM (BB/Wor rats), this loss was associated with a 9-fold increase of dystrophic neurites in the T2DM model. In addition, different from the T1DM model, protein levels of APP, β-secretase and Aβ were all increased in the T2DM rats, as were levels of hyperphosphorylated forms of tau. Collectively, the data show that accumulation of Aβ and hyperphosphorylated tau occurs in experimental diabetes. Interestingly, the changes were more severe in the T2DM model and appeared to be associated with insulin resistance and possibly, hypercholesterolemia (Li et al., 2007). To address the role of mitochondria as a functional link between both T2DM and AD, mitochondrial functions were assessed in 3xTg-AD mice that present with a combined Aβ and tau pathology, wild-type mice fed with 20% sucrose-sweetened water for 7 months (T2D mice), and wild-type mice that received plain water. The study found that compared to wild-type, mitochondria from 3xTg-AD and T2D mice presented with a similar impairment of the respiratory chain and oxidatitive phosphorylation system, a decreased capacity to accumulate calcium, ultrastructural abnormalities, and an oxidative imbalance, suggesting that the metabolic alterations associated with diabetes contribute to the development of AD pathology (Carvalho et al., 2012). In a follow-up study these mice were analysed behaviorally, by performing the open field, object recognition, Y-maze, and elevated plus maze tests. In addition, vascular functions were assessed. The T2D and AD mice showed comparable behavioral and cognitive impairments, i.e., increased fear and anxiety and decreased learning and memory abilities. Interestingly, both groups of mice presented with increased plasma markers of endothelial/vascular dysfunction and permeability of cerebral vasculature, and impaired mitochondrial enzymatic activities. Specifically, a decrease in complex IV activity was found in brain vessels and synaptosomes from T2D mice, whereas in the 3xTg-AD mice, this decrease was only seen in synaptosomes. The study furthermore found a significant increase in Aβ levels in the cortex and hippocampus of T2D mice supporting the notion that T2DM predisposes to cerebrovascular alterations, cognitive decline, and the development of AD (Carvalho et al., 2013). In another study, experimental DM was induced in FTLD tau transgenic mice and was found to exacerbate the tau pathology that characterizes this mouse model of AD (Ke et al., 2009). Together this shows that altered glucose levels have a dramatic effect on the tau and Aβ pathology that characterizes AD.

### Similar mechanisms of degradation and clearance of Aβ and amylin

Levels of Aβ and amylin are determined by the net effect of (1) their production, and (2) their degradation and clearance. Aβ-degrading peptidases *in vivo* are neprilysin (NEP) and Insulin Degrading Enzyme (IDE). For IDE, additional substrates were identified, such as amylin or insulin (Qiu and Folstein, 2006). Enhanced IDE activity correlates well with decreased Aβ levels in brains of IDE/APP double transgenic mice (Leissring et al., 2003), and IDE shows a decreased degrading activity of Aβ in AD compared to control brains (Leissring et al., 2003). *In vivo*, IDE substrates compete with each other, and this imbalance may contribute to the pathogenesis of AD and T2DM (Qiu and Folstein, 2006). Interestingly, mutations in *IDE* cause human T2DM-like symptoms (Farris et al., 2004). As far as neprilysin is concerned, there are conflicting data as to how this peptidase may impede islet amyloid formation. One study showed that it prevented fibrillization of amylin by cleaving it at up to six sites and that β-cells were protected from toxicity when amylin was added either exogenously or produced endogenously (Guan et al., 2012). Another study used mass spectrometry to conclude that islet amyloid formation was impeded by inhibition of fibril formation rather than by actual peptide degradation (Zraika et al., 2010). However, irrespective of the mode of action, it is fair to say that boosting the activities of these clearing enzymes is likely to be a valid strategy in reducing the pathological changes in both AD and T2DM.

### Toxicity of Aβ and amylin

Similar to Aβ, amylin can induce apoptotic cell-death (Hiddinga and Eberhardt, 1999). When we incubated hippocampal and cortical primary neurons with either non-aged or aged preparations of Aβ 42 and human amylin, based on the aggregation state of rat and human amylin as well as that of Aβ 42, we expected that the two latter molecules would exert a similar degree of toxicity. We found that in 20 DIV (days *in vitro*) hippocampal cultures, non-aged Aβ 42 was able to illicit toxicity at 5 μ M, and this effect was enhanced by aging of Aβ 42, whereas aged preparations of Aβ 42 were already significantly toxic at 0.5 μ M, compared to the PBS control (Lim et al., 2008). We observed a similar dose-dependency in the human amylin-treated cells, revealing enhanced neurotoxicity of aged compared to non-aged human amylin preparation. Different from human amylin and Aβ 42, rat amylin was not toxic to hippocampal neurons at either concentration tested. This suggested to us that a specific receptor-peptide interaction may be involved in neurotoxicity. Our data is consistent with studies in HeLa and β-cells in which human, but not non-amyloidogenic mouse amylin induced an aggregation state-dependent apoptosis (Ritzel and Butler, 2003). When we exposed cortical neurons to either Aβ 42 or human amylin, we found that, different from hippocampal cultures, these were generally less susceptible. Similar to Aβ 42, aging of human amylin enhanced its neurotoxic properties to a degree similar to what was found for hippocampal cultures. Together, this suggests that hippocampal neurons are more susceptible to the neurotoxic effects of both human amylin and Aβ 42, indicating selective vulnerability. We also found that rat amylin, which does not form fibrils, while not being toxic to hippocampal neurons, surprisingly, it was to cortical neurons (Lim et al., 2008).

While it is still not fully clear to which cellular components Aβ binds in order to exert its toxicity it appears unlikely that there are just one or two cognate receptors, or that there are only one or two assembly forms of the peptide that can induce neuronal dysfunction; in fact, Aβ is likely to bind to different components of neuronal and non-neuronal plasma membranes and thereby induces complex patterns of synaptic dysfunction and network disorganization (Mucke and Selkoe, 2012). As far as amylin is concerned, this molecule binds to receptors (such as the amylin receptor) but at the same time, using a combination of x-ray diffraction and circular dichroism, it was found that the β-aggregates of amylin can also bind directly on the surface of lipid bilayers, without penetrating into the bilayer structure (Lee et al., 2012).

### Proteomics sheds light on amylin's and Aβ's damaging effects on mitochondrial functions

In our hands, assessing changes to the proteome in the presence of protein aggregates composed of either Aβ, amylin, or tau has been particularly insightful. We entered the field by performing a proteomic analysis on fractionated brain extracts from tangle-forming P301L tau mutant pR5 mice, a model of the tau pathology of FTLD and AD (David et al., 2005). This approach identified mainly metabolism-related proteins including mitochondrial respiratory chain complex components, antioxidant enzymes, and synaptic proteins as being deregulated. As this indicated that only a limited number of cellular functions were impaired, this prompted us to functionally validate these proteomics findings. We were able to demonstrate a mitochondrial dysfunction in the pR5 mice, associated with higher levels of reactive oxygen species (ROS) and an up-regulation of antioxidant enzymes. Furthermore, the pR5 mitochondria displayed an increased vulnerability toward fibrillar Aβ peptide (David et al., 2005). We found that tau mainly impairs complex I of the respiratory chain, whereas Aβ (as shown in Aβ plaque-forming APP transgenic mice) mainly impairs complex IV (Hauptmann et al., 2008).

In many cellular systems including pancreatic β-cells it has been shown that nutrient fluctuations and insulin resistance increase proinsulin synthesis in β-cells beyond the capacity for folding of nascent polypeptides within the endoplasmic reticulum (ER) lumen, thereby disrupting ER homeostasis and triggering the “unfolded protein response (UPR)” (Song et al., 2008). This happens in part through the UPR-induced transcription factor C/EBP homologous protein (CHOP). In both genetic and diet-induced models of insulin resistance, CHOP deficiency improved β-cell ultrastructure and promoted cell survival. In addition, isolated islets from *Chop* knockout mice displayed increased expression of UPR (*BiP*, *Grp94*, *Fkbp11*, and *p58*^*IPK*^) and oxidative stress response genes (*Sod1*, *Sod2*, *Gpx1*, *Pparg*, and *Ucp2*), and reduced levels of oxidative damage. These findings suggest that CHOP is a fundamental factor that links protein misfolding in the ER to oxidative stress and apoptosis in β-cells under conditions of increased insulin demand (Song et al., 2008). Transgenic expression of human amylin in islets induces β-cell apoptosis (Huang et al., 2007). Our proteomic and biochemical analysis of Aβ-injected pR5 mice, a model combining the tau and Aβ pathology of AD (Götz et al., 2001), also suggests an impaired “unfolded protein response” due to protein aggregation (David et al., 2006). Among the up-regulated proteins with roles in protein folding and stress response were ICDH, the proteasome subunit alpha type 3, and the mitochondrial Grp75. Down-regulated were several members of the peroxiredoxin family. Together, this supports the notion of an altered “unfolded protein response” in both AD and T2DM.

Regarding Aβ 's and amylin's toxicity, there is another level of complexity (Fodero-Tavoletti et al., 2011). In the case of Aβ, the peptide's size ranges from 38 to 43 amino acids; furthermore, Aβ is pyroglutaminated at its amino-terminus, which increases its toxicity (Schlenzig et al., 2012). Furthermore, Aβ and amylin assume various aggregation states that range from monomeric to oligomeric to fibrillar (Lesne et al., 2006). We found that different from monomeric Aβ, both fibrillar and oligomeric forms cause mitochondrial dysfunction (Eckert et al., 2008). For T2DM, evidence is accumulating that it is oligomeric amylin that participates in β-cell apoptosis, while the degree of toxicity exerted by fibrillar species is less clear (Matveyenko and Butler, 2006b; Lin et al., 2007). As the accumulation of monomeric species precedes the formation of oligomeric and fibrillar species (that are in an equilibrium), from a therapeutic point of view the safest strategy might be to reduce total amyloid levels rather than depleting distinct pools such as the oligomers.

At a proteomic level, T2DM and AD share a remarkably similar profile. This is impressively illustrated by an analysis of pancreatic islets that identified several novel proteins that are also associated with AD pathogenesis (Nicolls et al., 2003). Interestingly, in a proteomic study using Aβ-injected tau transgenic pR5 brains, we identified a significant subset of “islet” proteins to be deregulated upon Aβ injection, such as GRP78, valosin-containing protein, calreticulin, the HSP family or peroxiredoxin (David et al., 2006). Similarly, in the insoluble “formic acid” fraction of Aβ-treated P301L tau-transfected cells, we identified Insulin-like growth factor binding protein 2 precursor (IGFBP-2) as being up-regulated, again pointing at similarities between DM and AD. Together with work published by others (Hickey et al., 2009), this implies that similar proteins and pathways are activated by amylin and Aβ, respectively, in either pancreatic islets (T2DM) or neurons (AD).

To determine whether the same proteins or protein categories are deregulated by Aβ 42 and human amylin, we performed iTRAQ labeling followed by quantitative mass spectrometry of human SH-SY5Y neuroblastoma cells that had been treated for 5 days with aged preparations of Aβ 42, human amylin, rat amylin and the respective vehicle controls (Lim et al., 2010). When rigorous criteria were applied, Aβ 42 significantly deregulated 69 proteins, whereas human amylin deregulated 49 proteins. Remarkably, of these, 31 were shared in both the Aβ 42 and human amylin treatment groups. [Treatment with the non-amyloidogenic rat amylin identified only five proteins, of which one (midkine) was shared with human amylin]. Of the proteins that were significantly altered in both the Aβ 42 and human amylin groups, for all but one protein, the direction of change was the same. Significantly deregulated proteins were sorted manually using *Gene Ontology* (GO) into their respective categories. The functional group with the largest number of proteins that was significantly altered in response to both peptide treatments was associated with mitochondrial functions/energy metabolism and antioxidant activities, representing more than 25% of all significantly deregulated proteins. While the majority of mitochondrial/metabolic proteins was down-regulated, antioxidant proteins were mostly up-regulated, suggesting a typical cellular response to oxidative stress. Functional validation revealed that mitochondrial complex IV activity was significantly reduced after treatment with either Aβ 42 or human amylin, as was mitochondrial respiration. In comparison, complex I activity was only reduced after treatment with human amylin. Aβ 42 and human amylin, but not rat amylin, induced significant increases in the generation of ROS. Co-incubation of Aβ 42 and human amylin did not produce an augmented effect in ROS production, again suggesting common toxicity mechanisms (Lim et al., 2010).

Interestingly, mitochondria represent not only an indirect target; instead, in several studies Aβ has been localized to mitochondria (Caspersen et al., 2005) and shown to act directly on these organelles (Lustbader et al., 2004; Crouch et al., 2005) whose function it impairs (Keil et al., 2004; David et al., 2005; Eckert et al., 2008; Rhein et al., 2009). Among the mitochondrial proteins to which Aβ has been shown to bind is the enzyme amyloid-binding alcohol dehydrogenase (ABAD) (Yan et al., 1997; Takuma et al., 2005). ABAD converts estradiol to estrone, and its levels are critical as optimal estradiol levels are an important determinant of neuronal survival (Yang et al., 2007). To directly determine whether Aβ-induced toxicity is mediated by ABAD inhibition and to establish estradiol levels as a suitable readout, we employed the use of the ABAD inhibitor AG18051 *in vitro* (Kissinger et al., 2004). We found that AG18051 partially blocked the Aβ-ABAD interaction in a pull-down assay while it also prevented the Aβ 42-induced down-regulation of ABAD activity, as measured by levels of estradiol, a known hormone and product of ABAD activity. Furthermore, AG18051 significantly reduced Aβ 42 toxicity, as measured by LDH release and ROS levels in SH-SY5Y human neuroblastoma cells. Guided by our previous finding of shared aspects of the toxicity of Aβ and human amylin, we determined in SH-SY5Y cells whether AG18051 would also confer protection from human amylin toxicity. We found, however, that the inhibitor conferred only partial protection from human amylin toxicity indicating partly shared yet still distinct patho-mechanisms of the two amyloidogenic agents (Lim et al., 2011).

### Common treatment strategies?

The biochemical commonalities between T2DM and AD would imply that common treatment strategies could be pursued. Among the potentially suitable strategies are vaccinations that have been successful in mice, targeting both Aβ and tau (Götz et al., 2012). Unfortunately, the clinical trials using anti-Aβ antibodies basically failed to meet their primary end points (Karran, 2012), while tau-based strategies have not yet been tested in a clinical setting. These failures are disappointing and bear the danger that the pharmaceutical industry will gradually retreat, however, there is a lot that can be learned from these failures, such as antibody dosing, design and route of administration, and it is certainly too early to give up. Because the immune response to amyloid oligomers is largely directed against generic epitopes that are common to amyloid oligomers of many different proteins and independent of a specific amino acid sequence, it is even possible to use an oligomer with a random sequence (3A) that reacts with the oligomer-specific antibody A11 as an effective immunogen (Rasool et al., 2012). Aβ plaque-forming Tg2576 mice were immunized with an Aβ oligomer, a human amylin oligomer, Aβ fibrils, and 3A oligomers. Although the human amylin oligomer and 3A oligomer mimics-vaccinated mice showed a relatively low titer as compared to the Aβ oligomer- and Aβ fibril-vaccinated groups, they retained their efficacy in preventing cognitive deficits and amyloid deposition. The data would suggest that an effective targeting of toxic species rather than the overall extent of the elicited immune response may be relevant (Rasool et al., 2012).

The effectiveness of antioxidants in modulating oxidative stress in AD has been widely explored. The antioxidants that have been shown to protect against Aβ-induced oxidative stress include vitamin E, various polyphenols, such as quercetin and resveratrol, α-lipoic acid, and curcumin (a planar biphenolic compound found in the Indian spice turmeric) (Pocernich et al., 2011). Curcumin has been shown to reduce Aβ pathology in various mouse models (Yang et al., 2005), by a mechanism that includes attenuating the maturation of APP in the secretory pathway (Zhang et al., 2010). Less data are available on the effect of curcumin in ameliorating the toxic effects of amylin. In a recent study it was found that curcumin alters amylin's misfolding and protects from cellular toxicity at physiologically relevant concentrations. Using electron paramagnetic resonance spectroscopy, thioflavinT fluorescence and electron microscopy, curcumin was found to significantly reduce amylin fibril formation and aggregate formation by altering its morphology and structure. Yet β-cells were protected from exogenous amylin toxicity by curcumin only within a relatively narrow concentration range, because curcumin became cytotoxic at micromolar concentrations. In other models, curcumin failed to protect β-cells from amylin-induced apoptosis leading the authors to suggest that without further modification, the compound is unlikely to be therapeutically useful in protecting β-cells in T2DM from destruction (Daval et al., 2010). Another study found that curcumin delayed the self-assembly of amylin into NMR-invisible assemblies (Sparks et al., 2012). Interestingly, when a double N-methylated and hence conformationally constrained analogue of amylin was employed this blocked the cytotoxic self-assembly of Aβ (Rezaei-Ghaleh et al., 2011). Finally, using HEK293 cells that stably express the amylin receptor, both Aβ and human amylin were found to induce cell death at low micromolar concentrations. In the presence of human amylin, however, the effect of Aβ 42 was occluded, suggesting a shared mechanism of action between the two peptides. Moreover, the amylin receptor antagonist AC253 blocked toxic responses including cell death, in response to an activation of the amylin receptor by either human amylin itself, Aβ42, or upon their co-application. This suggests that the amylin receptor may represent a novel therapeutic target for the development of compounds in the treatment of neurodegenerative conditions such as AD (Fu et al., 2012). What might potential side effects be of amylin receptor antagonists? Since the amylin receptor regulates food intake and body weight and the amylin agonist davalintide (AC2307), a novel amylin-mimetic peptide, has been found to reduce food intake and increase weight loss (Mack et al., 2010), by extrapolation, amylin antagonists may cause increases in weight due to increased food uptake. To our knowledge, however, this has not been observed in the animal studies that either tested AC253 or related antagonists.

In conclusion research into both amylin and Aβ has proven mutually beneficial in designing treatment strategies that hopefully benefit both AD and T2DM.

### Conflict of interest statement

The authors declare that the research was conducted in the absence of any commercial or financial relationships that could be construed as a potential conflict of interest.
